# Use of Antimicrobial Nanoparticles for the Management of Dental Diseases

**DOI:** 10.3390/nano15030209

**Published:** 2025-01-28

**Authors:** Iris Xiaoxue Yin, Anjaneyulu Udduttulla, Veena Wenqing Xu, Kitty Jieyi Chen, Monica Yuqing Zhang, Chun Hung Chu

**Affiliations:** Faculty of Dentistry, University of Hong Kong, Hong Kong SAR 999077, China; irisxyin@hku.hk (I.X.Y.); anjaneya@hku.hk (A.U.); u3008489@connect.hku.hk (V.W.X.); chenjy679@mail.sysu.edu.cn (K.J.C.); monicayq@hku.hk (M.Y.Z.)

**Keywords:** nanoparticles, antimicrobial, antioxidant, silver, zinc oxide, chitosan, curcumin, dental diseases

## Abstract

Dental diseases represent a significant global health concern, with traditional treatment methods often proving costly and lacking in long-term efficacy. Emerging research highlights nanoparticles as a promising, cost-effective therapeutic alternative, owing to their unique properties. This review aims to provide a comprehensive overview of the application of antimicrobial and antioxidant nanoparticles in the management of dental diseases. Silver and gold nanoparticles have shown great potential for inhibiting biofilm formation and thus preventing dental caries, gingivitis, and periodontitis. Various dental products can integrate copper nanoparticles, known for their antimicrobial properties, to combat oral infections. Similarly, zinc oxide nanoparticles enhance the antimicrobial performance of dental materials, including adhesives and cements. Titanium dioxide and cerium oxide nanoparticles possess antimicrobial and photocatalytic properties, rendering them advantageous for dental materials and oral hygiene products. Chitosan nanoparticles are effective in inhibiting oral pathogens and reducing inflammation in periodontal tissues. Additionally, curcumin nanoparticles, with their antimicrobial, anti-inflammatory, and antioxidant properties, can enhance the overall performance of dental materials and oral care products. Incorporating these diverse nanoparticles into dental materials and oral care products holds the potential to significantly reduce the risk of infection, control biofilm formation, and improve overall oral health. This review underscores the importance of continued research and development in this promising field to realize the full potential of nanoparticles in dental care.

## 1. Introduction

Nanoscience is the study of manipulating materials at the atomic, molecular, and macromolecular levels [1]. When applied to technology, this field is known as nanotechnology. Nanotechnology encompasses the design, production, characterization, development, and application of materials, devices, and systems through the manipulation of their shape and size at the nanometre scale [2]. This field has a broad range of applications across industry, health, the environment, and energy sectors, playing a crucial role in advancing nanomedicine and nanobiotechnology to improve human life [3,4].

Nanomaterials exhibit distinct physical, chemical, optical, mechanical, thermal, and biological properties compared to bulk materials, which are larger than microns. The use of nanomaterials in dentistry, particularly for the treatment of oral diseases, represents a pivotal area of inquiry within biological science and medical practice [5,6]. Nano dentistry is a burgeoning discipline that employs nanomaterials and nanotechnology for the diagnosis, treatment, and prevention of dental diseases [7,8].

Nanoparticles are the building blocks of nanomaterials with dimensions ranging from 1 to 100 nm [9]. Their high surface area-to-volume ratio, shape, dimensions, and charge density make them suitable for a wide range of applications. The development of nanoparticles offers innovative and effective strategies for treating human-associated diseases [10]. Nanoparticles can be classified into three categories: single nanoparticles, aggregates, and agglomerates [11]. Each category exhibits different characteristics due to the nanoparticles’ distinct structures and the forces holding the particles together.

Single nanoparticles are individual particles incapable of further dissociation into smaller units. They possess unique properties due to their nanoscale dimensions, such as a high surface area-to-volume ratio, enhanced reactivity, and quantum effects. These properties make single nanoparticles particularly suitable for drug delivery applications.

Aggregates consist of nanoparticles fused together by strong forces, such as covalent or ionic bonds, creating large, stable, and rigid structures. These properties make aggregates suitable to produce composite materials with enhanced mechanical properties.

Agglomerates are clusters of nanoparticles held together by weak forces, such as van der Waals forces or electrostatic interactions, making them prone to dispersion or dissociation. The properties of agglomerates depend on the arrangement and interaction of the constituent nanoparticles. Drug delivery uses them, achieving controlled release by dissolving the weak forces holding the agglomerate together [12]. The formation of aggregates and agglomerates occurs due to the accumulation of nanoparticles with high surface energy, which increases their size and limits their diffusion activity. Their biological and physicochemical properties vary depending on their structure and size [13].

Nanoparticles’ high surface area, shape, and charge allow them to interact easily with biological systems, enhancing their reactivity. These properties also give us many ways to change nanoparticles so that drugs can be delivered more precisely to specific sites, spread more evenly in the body, and cause less harm [14]. Nanoparticles can penetrate tissues and cells due to their smaller size. However, their penetration is not solely dependent on their size, but also on their shape and charge [15]. These characteristics enable the use of nanoparticles in the medical field for diagnostics, imaging, and other applications [16]. Due to their advantageous properties, nanoparticles gained significant momentum in medicine, but toxicity, drug delivery limitations, and uneasy handling limit their expansion and improvement of functions [17].

Researchers are also exploring nanoparticles as therapeutic agents in tissue engineering and the regenerative field to enhance treatment efficiency and reduce side effects. Additionally, researchers can use nanoparticles for early detection of oral cancer. Researchers use nanosensors to identify cancer biomarkers. Additionally, they use nanoparticles for targeted drug delivery to treat oral cancer more effectively while minimizing side effects.

The usage of nanosized particle-based therapeutics has been significantly rising in dentistry due to their antimicrobial and antioxidant properties. The nanoparticles have the potential to control planktonic bacteria growth and further biofilm formation [18]. People often consume natural antioxidants such as vitamin E and ascorbic acid through food or dietary supplements to combat oral-associated inflammatory diseases. However, the potential efficacy of these natural antioxidants in combating oxidative stress is often insufficient [19,20,21]. Recently, certain nanoparticles have garnered significant attention for their role in oral disease treatment [22].

However, the use of nanoparticles for the management of oral disease encounters some challenges. The potential toxicity of nanoparticles to human cells, organs, and the whole body is a significant concern, necessitating thorough biocompatibility testing. Additionally, the oral environment is a complex and dynamic ecosystem that involves a delicate balance between various biological, chemical, and mechanical factors. Ensuring the stability of nanoparticles in the oral environment can be challenging. Thus, researchers develop different delivery systems for the targeted use of nanoparticles in dentistry, treating various oral diseases and infections more effectively. Researchers can load these delivery systems with antimicrobial nanoparticles and deliver them directly to the infection site, thereby strengthening the nanoparticles’ stability, reducing the risk of antibiotic resistance, and minimizing side effects [3,4]. This review provides an overview of the use of antimicrobial nanoparticles in the management of dental diseases.

## 2. Mechanism of Action of Nanoparticles in Preventing Dental Diseases

**Figure 1:**
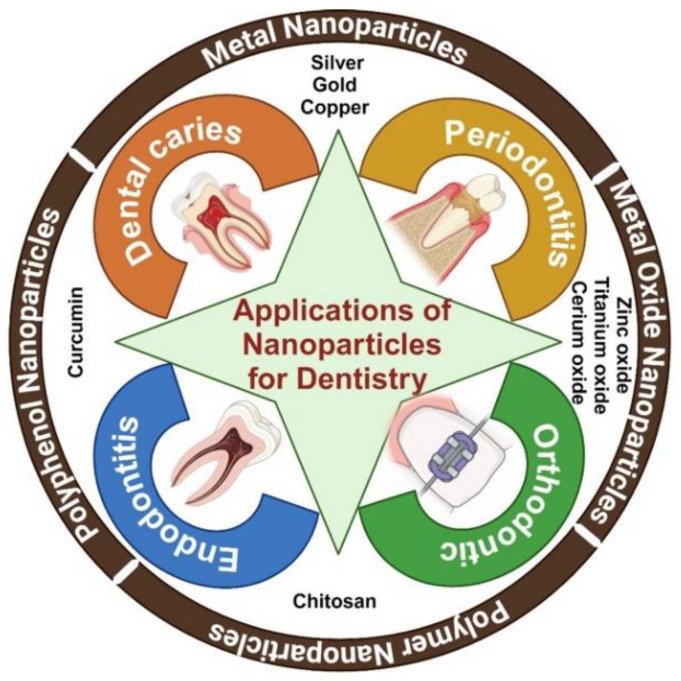
Applications of nanoparticles in dentistry.

Researchers have extensively studied nanoparticles for use in dentistry. Figure 1 shows the applications of nanoparticles in dentistry. Researchers have been studying the use of antimicrobial nanoparticles, including metals like silver, gold, and copper; metal oxides such as zinc oxide, titanium dioxide, and cerium oxide; polymers like chitosan; and polyphenols such as curcumin, for the prevention and treatment of oral diseases. Antimicrobial nanoparticles can be incorporated into dental fillings, sealants, and varnishes to inhibit the growth of cariogenic bacteria. They can also be incorporated into orthodontic appliances to prevent dental caries. Antimicrobial nanoparticles can be used in mouthwashes, gels, and local delivery systems to manage periodontal pathogens. They can be included in root canal treatments to ensure thorough disinfection of the root canal system.

**Figure 2:**
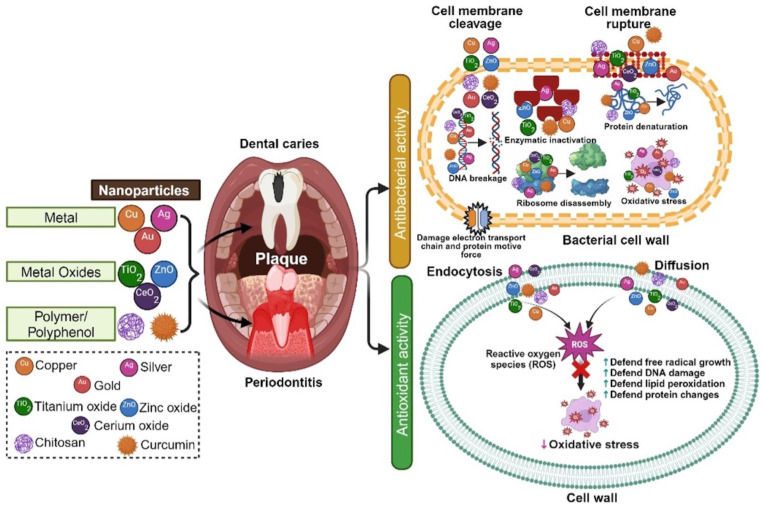
Mechanism of action of nanoparticles for prevention of dental diseases.

Nanoparticles are increasingly being explored for their potential in managing dental diseases due to their superior antimicrobial and antioxidant properties. Figure 2 shows the mechanism of action of nanoparticles in preventing oral diseases. The nanoparticles have the potential to control planktonic bacteria growth and further biofilm formation. While some nanomaterials have inherent antibacterial properties, reducing their physical dimensions to the nanoscale level could enhance their antibacterial activity by facilitating easier interaction and penetration with bacteria [18]. The antimicrobial mechanisms of nanoparticles have not been fully understood. There are several actions proposed to explain the antimicrobial mechanisms. Nanoparticles can interact with microbial cell membranes, causing structural damage and leakage of cellular contents. Some nanoparticles induce oxidative stress in microbial cells, leading to cell death. They can bind to microbial proteins and DNA, inhibiting their function and replication. In addition, nanoparticles can penetrate and disrupt biofilms, which are protective layers formed by microbial communities.

Oxidative stress, a condition characterized by an excess of oxidants, arises from an imbalance between free radical production and the antioxidant defenses of the human body. This condition leads to the generation of reactive oxygen species and free radicals, which are highly unstable and reactive molecules capable of damaging cellular metabolism, functions, and tissues [19].

In the context of periodontitis and dental caries, bacteria produce toxins, detrimental metabolites, and histolytic enzymes that can trigger the host’s immune response. The activated immune cells then release excessive amounts of reactive oxygen species and proinflammatory cytokines. This cascade of cytokines and reactive oxygen species perpetuates the inflammatory response, leading to damage to DNA, proteins, cells, and tissues [20]. Therefore, the treatment of chronic periodontitis and dental caries requires antioxidant-based therapeutics to mitigate the inflammatory response. These antioxidant materials function by inhibiting the activity of reactive oxygen species and free radicals, stabilizing them by providing electrons. Nanoparticles, whether in the form of metals, metal oxides, or functionalized with natural antioxidant materials, are used to prevent oxidative stress by quenching free radicals and scavenging reactive oxygen species at sites affected by periodontitis and caries [22].

Antimicrobial nanoparticles are commonly utilized to enhance remineralization. The oral cavity, which is teeming with bacteria, has a biofilm on the tooth surface that continuously produces acid, resulting in ongoing mineral loss and a significant reduction in tooth structure. The integration of antibacterial nanoparticles with a remineralizing agent facilitates consistent and stable remineralization of dental hard tissue. Antibacterial nanoparticles enhance the quantity of remineralization by decreasing demineralization. When combined with nano-chitosan, a half-dose of bioactive glass was sufficient to generate equivalent quantities of new mineralized crystals [23]. Antibacterial nanoparticles, like chitosan, can be used as drug delivery systems to help antibacterial and remineralizing agents be released continuously. This stops demineralization and encourages mineral deposition. Nanoparticles that impede the proliferation of cariogenic bacteria and augment the hardness of dental hard tissue can decelerate demineralization and facilitate remineralization by neutralizing the acidic microenvironment and enhancing the resistance of hard tissue to acid.

The pellicle layer is a protein film that develops on the tooth surface shortly after cleaning. It functions as a protective barrier, diminishing enamel demineralization and offering a substrate for initial bacterial adhesion. The composition primarily includes proteins, glycoproteins, and lipids originating from saliva. The interaction of nanoparticles with the pellicle layer in the oral cavity is a critical factor influencing their efficacy. The flow rate and quality of saliva are likely to influence the dynamics of nanoparticle interactions with the pellicle. The protein corona formed on nanoparticles can affect their adhesion to the pellicle and, subsequently, their ability to interact with oral biofilms [24]. This interaction is crucial for the effectiveness of nanoparticles designed for applications such as antibacterial treatments and biofilm disruption. The pellicle layer generally exhibits a negative overall charge. Positively charged nanoparticles can attach to the pellicle layer and release antimicrobial agents, thus inhibiting bacterial colonization and biofilm development [25]. Moreover, the ability of nanoparticles to penetrate the dense crystalline structure of tooth enamel is limited; however, they may interact more readily with the underlying dentinal tubules, which are not as obstructive. This suggests that while the pellicle layer may act as a barrier, it also presents opportunities for nanoparticles to deliver therapeutic agents to deeper dental structures. Overall, the interaction of nanoparticles with the pellicle layer is a complex process that significantly impacts their clinical applications in dentistry. However, the antimicrobial effects of synthetic nanomaterials in complex clinically relevant biofilms, such as those on the pellicle, remain unclear. Further research is needed to elucidate the mechanisms underlying these interactions and their implications for nanoparticle efficacy in the oral cavity.

## 3. Use of Antimicrobial Nanoparticles for Dental Diseases

### 3.1. Silver Nanoparticles

Silver nanoparticles are among the most extensively studied nanomaterials in recent decades. Researchers have employed many strategies to produce silver nanoparticles, including chemical, physical, and biological methods [26]. The chemical reduction approach is the primary procedure, utilizing reducing agents such as sodium borohydride, ascorbic acid, or citric acid to transform silver ions into silver nanoparticles [26]. Alternative methodologies are under examination, encompassing physical and biological techniques such as laser ablation and eco-friendly synthesis employing plant extracts or microorganisms [27]. By controlling these processes, researchers can produce nanoparticles with varying sizes, shapes, and surface properties, which influence the dissolution efficiency of silver nanoparticles.

Smaller silver nanoparticles with spherical or quasi-spherical morphologies exhibit a greater propensity for silver release owing to their increased surface area. The dissolution efficiency of silver nanoparticles significantly influences their antimicrobial efficacy and biocompatibility. Silver nanoparticles have been studied for the treatment of oral diseases owing to their possible antibacterial properties [28]. In a moist environment such as the oral cavity, silver nanoparticles can continuously release silver ions, which is considered one of the mechanisms for killing microbes. Silver nanoparticles engage with microbial cell walls, proteins, and nucleic acids, leading to cellular impairment and death [29]. They can induce the production of reactive oxygen species, including superoxide radicals, hydrogen peroxide, and hydroxyl radicals, which cause oxidative stress and damage to microbial cells. Furthermore, silver nanoparticles can interact with negatively charged microbial cell membranes, leading to membrane breakdown, nutritional depletion, and eventual cell death Their nanoscale size allows them to penetrate microbial cells and bind to intracellular proteins, enzymes, and DNA, inhibiting their functions and causing cell death [30].

To improve the quality of dental appliances for managing dental diseases, researchers have developed antimicrobial materials incorporating silver nanoparticles. Dental caries result from the demineralization of enamel and dentin due to acidogenic bacteria, including *Streptococcus mutans*. Silver nanoparticles can be incorporated into mouthwashes and toothpastes to diminish bacterial adhesion and impede plaque formation [31]. When combined with sodium fluoride, silver nanoparticles can inhibit bacterial growth and facilitate the remineralization of demineralized dentine, thereby preventing caries. Furthermore, dental restorative materials, including composite resins, glass ionomer cements, and dental adhesives, may integrate silver nanoparticles to inhibit secondary caries [32].

Periodontal disease primarily results from the accumulation of bacterial biofilms and the host immune response. Gram-negative bacteria like *Porphyromonas gingivalis*, which are the main cause of periodontal infections, are inhibited by silver nanoparticles. Researchers have integrated silver nanoparticles into guided tissue regeneration membranes to diminish bacterial adherence and penetration, thereby enhancing the success of intrabony defect treatments. Moreover, silver nanoparticles exhibit anti-inflammatory properties by regulating the concentrations of inflammatory cytokines and growth factors. Periodontal dressings infused with silver nanoparticles can be utilized for the treatment of gingival wounds [33].

Peri-implantitis is an inflammatory disorder that impacts the tissues surrounding dental implants, resulting in bone resorption and potential implant failure. Silver nanoparticles can be coated on implant surfaces to provide antimicrobial properties and reduce the risk of peri-implantitis without significant cytotoxic effects on osteoblastic cells [34].

Silver nanoparticles exhibit antibacterial effectiveness against anaerobic endodontic pathogens, including *Enterococcus faecalis* and *Candida albicans*. They can be used as a substitute for sodium hypochlorite in root canal irrigation to disinfect the root canal system. Endodontic materials like gutta-percha and mineral trioxide aggregate can incorporate silver nanoparticles to enhance the success of endodontic treatment [35]. Moreover, adding silver nanoparticles to acrylic resin can also prevent denture stomatitis by stopping oral pathogens from taking advantage of opportunities [36]. Although silver nanoparticles may offer advantages in the management of dental diseases, specific risks and concerns must be considered. Silver nanoparticles may demonstrate cytotoxic effects on human cells, potentially impairing tissue healing and regeneration. Elevated levels of silver nanoparticles or extended exposure may result in cellular damage, oxidative stress, and inflammation [37]. Consequently, optimizing the concentration and dimensions of silver nanoparticles in dental applications is essential to guarantee their safety and biocompatibility.

The extensive application of silver nanoparticles generates apprehensions regarding their possible environmental consequences. Silver nanoparticles can infiltrate the environment via wastewater systems, resulting in the accumulation of silver in aquatic ecosystems, soil, and living organisms [38]. The enduring consequences of this accumulation on ecosystems and human health remain uncertain. It is imperative to formulate strategies for the secure disposal and recycling of dental materials containing silver nanoparticles to mitigate their environmental impact.

In conclusion, silver nanoparticles possess considerable promise for the treatment of dental problems owing to their antibacterial characteristics and capacity to improve dental materials. However, to address the challenges related to their cytotoxicity, environmental impact, and optimal application methods, further research is necessary. By overcoming these challenges, silver nanoparticles could become a valuable tool in improving oral health and advancing dental treatments.

### 3.2. Gold Nanoparticles

Gold is a promising and stable nanomaterial for medical applications due to its unique physicochemical properties, biocompatibility, electrical conductivity, and optical characteristics. Gold nanoparticles possess antibacterial, antifungal, and anticancer properties, rendering them significant for the treatment of dental diseases such as dental caries, peri-implantitis, and oral cancer [39].

The antibacterial and antifungal activities of gold nanoparticles depend on their size, shape, and surface area per unit volume. Additionally, these nanoparticles serve as excellent vehicles for delivering antibiotics, drugs, and genes to specific sites for enhanced biological action. Although the antibacterial activity of gold nanoparticles is relatively weak, their ability to kill bacteria through direct cell-wall interactions is significant. Gold nanoparticles are independent of reactive oxygen species and exhibit minimal cytotoxicity to mammalian cells [40].

Researchers have demonstrated the antibacterial activity of various morphology-based gold nanoparticles, such as spherical, nanorod, and core/shell, in preventing caries caused by *S. mutans*. They reported that all types of gold nanoparticles exhibited good antibacterial activity, with no significant differences among them [41]. Another study reported the antibacterial activity of gold nanoparticles against periodontal disease-causing bacteria like *Streptococcus oralis*. They found that gold nanoparticles at 100 ppm exhibited a similar antibacterial effect to chlorhexidine compounds [42].

Researchers also developed gold–titanate complexes to eradicate cariogenic bacteria such as *Lactobacillus casei* and *S. mutans*. They confirmed that the developed complex enhanced antibacterial activity and significantly inhibited bacterial growth [43].

The use of gold nanoparticles for treating infections is continually advancing. However, extensive research, including clinical trials, is necessary to ensure their safety and long-term effectiveness.

### 3.3. Copper Nanoparticles

Advancements in nanotechnology in recent years have led to the development of various copper-based nanoparticles with antibacterial properties for managing oral disorders [44]. The superior antibacterial capabilities of copper-based nanoparticles are primarily due to their high surface-area-to-volume ratio [45]. Copper-based nanoparticles demonstrate reduced toxicity to human cells in comparison to silver nanoparticles [46]. Additionally, copper nanoparticles are stable and inexpensive to synthesize because copper is readily accessible [47]. Copper nanoparticles have demonstrated antibacterial properties against a wide range of microorganisms. Various applications have utilized them to treat oral diseases, either alone or in combination with other materials. Copper nanoparticles have been integrated into topical agents, dental cements, and dental adhesives to confer antimicrobial properties for the management of dental caries. One in vitro study reported that glass ionomer cement with copper nanoparticles exhibited low cytotoxicity to cells and demonstrated antibacterial effects against *S. mutans* and *Streptococcus sanguinis* [48]. A further study demonstrated that a topical agent of silver diamine fluoride and bio-glass infused with copper nanoparticles improved antibacterial efficacy against *S. aureus* and *S. mutans* without diminishing cytotoxicity [49]. In a separate investigation, copper, silver, and metronidazole nanoparticles were incorporated into glass ionomer cement, effectively inhibiting the growth of *S. mutans* and *S. aureus* [50].

Researchers have also incorporated zinc oxide and copper nanoparticles into dental adhesives for dentine bonding [51]. They found that the addition of zinc oxide and copper nanoparticles increased the antimicrobial activity and ultimate tensile strength of the adhesive without affecting the bond strength between the adhesive and dentine or causing nano-leakage. Another group developed an adhesive with fluoride-containing zinc oxide and copper oxide nanocomposites, which exhibited superior antibacterial effects against *S. mutans* compared to conventional adhesives.

Researchers also developed antibacterial graphene oxide-copper nanocomposites with sustained release of copper nanoparticles [52]. These nanocomposites significantly reduced the biomass of *S. mutans* biofilms, potentially modified their structure, hindered the production and transportation of exopolysaccharides, and disrupted the expression of genes associated with exopolysaccharide synthesis [52].

Copper nanoparticles also exhibit excellent antibacterial properties for the prevention of periodontal diseases. Researchers have designed sponges and gel spheres of chitosan loaded with copper nanoparticles, effectively inhibiting the growth of *Aggregatibacter actinomycetemcomitans* and supporting the development of localized periodontal therapies [53]. Additionally, scientists synthesized copper nanoparticles using Cupressus macrocarpa extract and investigated their antibacterial effectiveness against *Micrococcus luteus*, *Bacillus subtilis*, and *Pseudomonas aeruginosa* to prevent periodontitis [54]. Copper nanoparticles can promote bone growth to treat periodontal disease. A research group developed a novel chitosan-modified copper sulfide nanocluster, which exhibited antibacterial activity against *Fusobacterium nucleatum* and showed potential for treating periodontitis and promoting alveolar bone growth [55].

Copper nanoparticles have also been coated onto the surface of dental implants to treat peri-implantitis. In one study, a research group examined the properties of copper-coated dental implants and found that the coated copper nanoparticles exhibited strong antibacterial activity against *Porphyromonas gingivalis*, thereby preventing infection in the area surrounding dental implants. Another study investigated copper-doped mesoporous bioactive glass nanoparticles and found that these nanoparticles exhibited angiogenic and antibacterial properties when coated on dental implants [56]. Additionally, copper oxide nanoparticles can be utilized as potential corrosion inhibitors for dental implants to increase their corrosion resistance [57].

Although copper nanoparticles are effective in a wide range of oral disease control, most publications have been in vitro studies with relatively few in vivo studies available. More in vivo studies are essential to validate the effectiveness and safety of copper nanoparticles in dental applications. This additional research will help determine the long-term outcomes and potential side effects of copper-based treatments in real-world clinical settings.

In summary, copper-based nanoparticles have shown considerable promise for managing various oral diseases due to their excellent antibacterial properties, stability, and cost-effectiveness. Their incorporation into dental materials such as cements, adhesives, and topical agents can enhance the antimicrobial efficacy of these products while maintaining biocompatibility. However, to fully realize the potential of copper nanoparticles in dental care, further extensive in vivo research is required to confirm their clinical efficacy and safety. Addressing these challenges will pave the way for the broader adoption of copper nanoparticles in dental treatments.

### 3.4. Zinc Oxide Nanoparticles

Zinc oxide nanoparticles are among the most widely produced nanomaterials worldwide [58,59]. The United States Food and Drug Administration has designated zinc oxide nanoparticles as Generally Recognized as Safe. In dentistry, these nanoparticles have extensive applications due to their unique optical, catalytic, mechanical, biological, photothermal, electrical, and magnetic properties [60,61]. These properties can be tailored through modifications in synthetic conditions, the addition of other inorganic substances, and alterations in size [62].

Zinc oxide nanoparticles are widely adopted in dentistry due to their antimicrobial, anticancer, and remineralization capabilities. Researchers are exploring their use across almost all branches of dentistry, including restorative dentistry, endodontics, periodontics, prosthodontics, orthodontics, oral medicine, oral maxillofacial surgery, dental implantology, and preventive dentistry [63]. One major reason for adopting zinc oxide nanoparticles in dentistry is their ability to control microorganisms and their activities.

Zinc oxide nanoparticles have been used as anti-caries agents [64]. A laboratory study reported that zinc oxide nanoparticle varnish significantly inhibited S. mutans biofilm formation and reduced its acid production [65]. Composite resins containing zinc oxide nanoparticles significantly inhibited the growth of *S. mutans* and *Lactobacillus* [66]. Zinc oxide nanoparticles can be integrated into toothpaste, mouthwash, and varnish to inhibit dental cavities [65,67,68]. A study reported that titanium orthodontic screws coated with zinc oxide nanoparticles inhibited *Enterobacter aerogenes*, *S. aureus*, *S. mutans*, *E. faecalis*, *Escherichia coli*, and *C. albicans* [69]. Denture base resin with zinc oxide nanoparticles inhibited the biofilm growth of *S. mutans* and *C. albicans* [70]. Zinc oxide nanoparticles could also be adopted as coating materials to prevent periodontitis [71]. A hydrogel with zinc oxide nanoparticles was found to significantly inhibit the growth of *Streptococcus oralis*, *P. gingivalis*, *S. sanguinis*, and *Prevotella intermedia* [72]. Compared to antibiotics, zinc oxide nanoparticles exhibit antimicrobial activity at low concentrations and are non-toxic [73].

The precise mechanisms by which zinc oxide nanoparticles control microorganisms are not fully understood; however, some frequently reported mechanisms are summarized as follows. Zinc oxide nanoparticles have a high surface-to-volume ratio, which increases their reactivity and interaction with cells, leading to the destruction of bacterial cell integrity [74]. They release zinc ions that change the surface charge of the membrane and alter cell permeability, resulting in leakage of cellular contents [75]. The zinc ions also displace magnesium ions, inhibiting enzymatic activity and interfering with bacterial metabolism [63]. Moreover, zinc oxide nanoparticles generate reactive oxygen species such as superoxide radicals and hydroxyl radicals. These reactive oxygen species cause oxidative stress, consequently damaging bacterial cell membranes and internal structures [75]. Scientists are using zinc oxide nanoparticles to inhibit multiple drug-resistant bacteria because bacteria generally do not develop resistance to zinc oxide nanoparticles.

Zinc oxide nanoparticles also have potential as anticancer agents. Researchers have found that cancerous cells are more sensitive to zinc oxide nanoparticles than normal cells, and these nanoparticles can induce higher amounts of reactive oxygen species in cancerous cells than in normal cells. A study reported that zinc oxide nanoparticles induce toxicity toward human oral squamous carcinoma cells [76]. Another study found that zinc oxide nanoparticles inhibit the growth of human gingival squamous cell carcinoma by increasing intracellular reactive oxygen species and superoxide levels [77]. Additionally, zinc oxide nanoparticles can conjugate with acid-sensitive polymers, which can be taken up by cancer cells and then release drugs into the cancerous cells [78]. Researchers have also used zinc oxide nanoparticles to detect the salivary oral tumor biomarker interleukin-8 [79].

Researchers also use zinc oxide nanoparticles to enhance the remineralization of dental hard tissue [80]. They facilitate the uptake of calcium and phosphate ions and maintain the surface zone porosity for the movement of ions during remineralization [80]. Zinc ions induce apatite formation, which is crucial for the remineralization process [81]. Zinc nanoparticles can be cooperatively used with hydroxyapatite nanoparticles to promote the remineralizing effect. Research has indicated that toothpaste with zinc-carbonate hydroxyapatite nanoparticles offered superior remineralization on enamel compared to fluoride toothpaste in vivo. Zinc-carbonate hydroxyapatite nanoparticles can accelerate biomimetic mineralization on the enamel surface, mimicking the structure and composition of the natural enamel hydroxyapatite [82].

Some researchers have incorporated zinc oxide nanoparticles into polymethylmethacrylate to increase the flexural strength of denture base materials. Zinc oxide nanoparticles have been employed to modify titanium implant surfaces, facilitating osseointegration and improving the integration of implants with bone [83].

Zinc oxide nanoparticles are promising materials for managing oral diseases due to their non-toxic nature compared to other metal nanoparticles. However, it is important to note that high concentrations of zinc oxide nanoparticles can be toxic to human gingival fibroblasts and inhibit cell proliferation [84]. Although the literature does not report severe adverse effects of zinc oxide nanoparticles, most of the studies have been conducted in vitro or using animal models. Clinical trials are essential to substantiate the evidence of their effectiveness and safety for clinical treatment.

In summary, zinc oxide nanoparticles offer a wide range of applications in dentistry due to their unique properties and antimicrobial capabilities. They have shown potential in various dental treatments, including anti-caries agents, coatings for orthodontic screws and denture base resins, and materials for enhancing remineralization and osseointegration. Despite their promising potential, further clinical trials are necessary to confirm their long-term safety and effectiveness in human subjects. Addressing these challenges will pave the way for the broader adoption of zinc oxide nanoparticles in dental treatments, ultimately improving oral health outcomes and patient care.

### 3.5. Titanium Dioxide Nanoparticles

Researchers are increasingly interested in titanium dioxide nanoparticles due to their unique biological and physicochemical properties [85]. These nanoparticles can absorb photons with energy when exposed to ultraviolet light, leading to the formation of reactive oxygen species [86]. The high reactivity of these free radicals allows engineered titanium dioxide nanoparticles to produce light-induced biocidal effects against a wide range of pathogens [87,88]. Several studies have shown that titanium dioxide nanoparticles are bactericidal to *E. coli*, *S. aureus*, *S. mutans*, *S. sanguis*, and *C. albicans* [89,90,91,92]. These microorganisms are strongly associated with various oral infectious diseases, such as dental caries and periodontitis [93]. One laboratory study synthesized titanium dioxide nanoparticles that effectively killed multidrug-resistant strains of *P. aeruginosa*, which were completely resistant to cefepime and highly resistant to ceftriaxone, amikacin, and ciprofloxacin [94]. This study emphasized the superior capability of titanium dioxide nanoparticles over some antibiotics in treating multidrug-resistant bacteria.

Researchers have explored implant coatings with titanium dioxide nanoparticles to prevent infection [93,95,96]. Additionally, titanium dioxide nanoparticles can be used in combination with silver to achieve synergistic antibacterial effects [88,97]. Silver-loaded titanium dioxide nanoparticles have shown strong antibacterial properties against *S. aureus* and *E. coli* [98]. Beyond their anti-infection properties, an animal study demonstrated a significant increase in bond strength and osseointegration of dental implants modified with titanium dioxide nanotubes [99]. A separate study revealed that titanium surfaces with nano topography augmented osteoblastic activities while suppressing osteoclast differentiation and activity [100]. However, some researchers are concerned about the potential toxic effects and unintentional exposure risks associated with titanium dioxide nanoparticles. A review found elevated levels of titanium dioxide nanoparticles in vital organs such as the brain and liver after maternal exposure during pregnancy, leading to toxic effects and potential organ dysfunction [101]. Furthermore, the uptake of titanium dioxide nanoparticles may influence autophagic processes, which play a crucial role in maintaining cellular health and homeostasis [102]. While titanium dioxide nanoparticles offer promising antibacterial and osseointegration properties for dental applications, further research is needed to fully understand their potential toxic effects and ensure their safety. Addressing these concerns will be crucial for the broader adoption of titanium dioxide nanoparticles in clinical settings.

### 3.6. Cerium Oxide Nanoparticles

Cerium is a rare-earth metal that exists in dual oxidation states. Cerium dioxide nanoparticles, known as nanoceria, have a wide range of clinical applications due to their biocompatible, anti-inflammatory, antibacterial, antioxidant, antiapoptotic, and angiogenic properties [103]. Researchers are investigating nanoceria for treating bacterial-induced inflammation, such as peri-implantitis and periodontitis. The antibacterial mechanism of nanoceria does not depend on cell penetration. Instead, they provoke oxidative stress by generating reactive oxygen species, which harm RNA, DNA, and proteins. This reactive oxygen species generation is primarily due to the reversible conversion between the +3 and +4 oxidation states. Additionally, nanoceria exhibits antibacterial activity by indirectly interacting with bacteria, reacting with ions or reactive oxygen species, and causing harm by transferring surface ions to the bacterial cell membrane [104].

A study found dental composite resin with nanoceria had strong antibacterial activity against several pathogenic bacteria, including *S. mutans*, *S. aureus*, *Streptococcus mitis*, and *Lactobacillus* spp. [105]. Another study developed erythrocyte-imitating composites combining mesoporous polydopamine and nanoceria, confirming that these materials synergistically remove reactive oxygen species [106]. Researchers incorporated cerium nanoparticles into zeolitic imidazolate frameworks for periodontitis, enhancing their antibacterial and anti-inflammatory properties. They successfully inhibited biofilm formation by periodontal bacteria and evaluated the effects on inflammatory molecule secretion and macrophage polarization [107]. In a periodontal disease-induced animal model, nanoceria composites completely reduced local inflammation. Nanoceria offers solutions to several dental problems that conventional antibacterial agents cannot address. However, the key issue of nanoceria toxicity needs to be addressed.

### 3.7. Chitosan Nanoparticles

Chitosan is a naturally occurring cationic polyelectrolyte copolymer derived from chitin [108]. Chitosan possesses intrinsic antibacterial properties, which make it suitable for various applications, including wound dressings, food preservation, and pharmaceuticals [109]. Chitosan nanoparticles are used for drug delivery for endodontic therapy, oral hygiene products for caries prevention, and implant coatings for prevention of peri-implantitis [110,111,112]. Chitosan nanoparticles are also used in periodontal treatment and oral surgery to prevent infection [113,114]. Researchers used chitosan nanoparticles for direct pulp capping to enhance cell viability and promote wound healing [115]. Chitosan nanoparticles possess both antioxidant and free radical scavenging properties [116]. An animal study showed Chitosan nanoparticles could alleviate osteoblast dysfunction and apoptotic injury in rat osteoblasts and promote impaired implant osseointegration [117]. The antimicrobial mechanism of chitosan is unknown and relatively weak compared to other antimicrobial nanoparticles [118,119,120]. Thus, chitosan nanoparticles are always cooperatively used with other antimicrobial materials. The silver-chitosan nanoparticle demonstrated a significant inhibition on the growth of *S. mutans* and *P. gingivalis.* These two bacteria are major dental pathogens for dental caries and periodontitis. Additionally, chitosan nanoparticles effectively prevented the adhesion of these bacteria and reduced biofilm formation by downregulating quorum-sensing molecules [121]. They inhibited the growth of periodontal pathogens, specifically *P. gingivalis* and *A. actinomycetemcomitans*, and modulated the inflammatory response. This finding suggests that chitosan could be beneficial in the prevention and treatment of periodontal inflammation and infection [122]. A study demonstrated that chitosan/hydroxyapatite nanocomposite coatings enhanced the apatite formation [123].

### 3.8. Curcumin Nanoparticles

Curcumin is a naturally occurring phenolic pigment substance extracted from the roots of curcuma longa [124]. It has antibacterial, anti-inflammatory, and antioxidant properties [125,126]. The antibacterial action of curcumin may be attributed to its ability to alter cell membrane permeability and interact with the prokaryotic filamenting temperature-sensitive mutant Z protein [124,127,128]. Additionally, curcumin may interfere with the repair process of bacterial DNA [129,130]. The anti-inflammatory property has been adopted in the treatment of systematic disorders like diabetes and Alzheimer’s disease [131,132]. The antioxidant of curcumin neutralizes harmful reactive oxygen species and free radicals in cells, thereby preventing cellular damage [133]. Despite its potential pharmaceutical benefits, the effectiveness of curcumin has been limited by its poor solubility and inconsistent bioavailability [134]. Nano-synthetic techniques have been developed to address these issues, resulting in the development of curcumin nanogels with superior antibacterial efficacy than curcumin [135]. Researchers developed nanomaterials using curcumin with other antibacterial agents to inhibit various bacteria, including *P. aeruginosa*, *S. aureus*, *E. coli*, and *B. subtilis* [119,136,137]. A clinical trial showed curcumin nanoparticles are more effective than ornidazole gel in treating severe periodontitis [138]. Table 1 shows the biological properties, potential applications, and limitations of some common nanoparticles used in dentistry.

## 4. Challenges of Use of Nanoparticles in Dentistry

Despite the outstanding potential of nanoparticles for treating and managing dental diseases, their extensive long-term applications in dentistry must acknowledge and address several limitations. Manufacturing, biological, ethical, and commercialization aspects categorize the key challenges associated with the use of nanoparticles [112].

The first challenge is manufacturing limitations. Producing nanoparticles with consistent quality and size distribution can be difficult. This variability affects the quality and properties of nanoparticles, thus impacting their effectiveness in treating dental diseases [113].

The second challenge involves biological limitations. Nanoparticles can potentially cause toxicity to human cells and tissues, leading to adverse health effects. Some nanoparticles may be cytotoxic, genotoxic, or even carcinogenic, causing harm to oral tissues, especially when used at high concentrations or for extended periods [114]. The biodistribution and clearance of nanoparticles in the body are not yet fully understood. Prolonged exposure to nanoparticles may lead to their accumulation in certain organs or tissues, which could cause unintended side effects. Due to their nanosized nature, nanoparticles can easily enter human tissues, cross the blood–brain barrier, and reach the lungs, posing potential health risks [115].

The third challenge pertains to ethical limitations. The use of nanoparticles in dentistry raises ethical concerns, particularly regarding dosage, informed consent, patient privacy, and potential health risks. The safety and efficacy of nanoparticle-based treatments must be thoroughly evaluated before clinical approval. The regulatory approval process for new nanomaterials can be lengthy and complex, potentially delaying the availability of these treatments for patients [116].

The fourth challenge involves commercialization limitations. Developing and manufacturing nanoparticle-based treatments can be expensive, which could limit their accessibility for patients, particularly in countries with weaker economies and less robust research and development infrastructures [117]. Researchers are actively exploring strategies to overcome these challenges and facilitate the use of nanoparticles for the prevention and treatment of oral diseases.

Nanoparticles are revolutionizing dentistry by offering enhanced treatment options. Their unique properties, such as tiny size, large surface area-to-volume ratio, and high reactivity, make them ideal for various applications. Nanoparticles such as silver, copper, zinc oxide, titanium dioxide, chitosan, and curcumin have been extensively studied for their antibacterial and antioxidant properties in dental applications. These nanoparticles have the potential to control bacterial growth, inhibit biofilm formation, and alleviate oxidative stress.

The incorporation of nanoparticles into various dental materials, such as composite resins, glass ionomer cements, dental adhesives, and implants, has shown promise in preventing caries and periodontal disease, promoting bone growth, enhancing remineralization, and improving osseointegration. While nanoparticles offer significant benefits, their long-term effectiveness and safety require further investigation.

The development of safe and effective nanoparticle-based therapeutics could revolutionize the way we treat and manage oral diseases, leading to better oral health outcomes worldwide. In conclusion, while nanoparticles have promising potential in dentistry, addressing manufacturing, biological, ethical, and commercialization challenges is crucial for their successful integration into clinical practice.

## 5. Future Directions

Nanoparticles are gaining significant attention in dentistry; however, their applications require more evidence-based studies to fully realize their potential. Researchers, with the support of clinicians and industries, are actively developing innovative nanoparticles for dental applications. Some researchers believe that smart nanoparticles, such as stimuli-responsive and electroactive nanoparticles, could offer promising treatments for dental diseases.

Stimuli-responsive nanoparticles, which can be combined with drugs, genes, and proteins, have the potential to treat antimicrobial-resistant infections, deliver drugs precisely to target sites, and be used in photodynamic therapy applications. These nanoparticles can provide diagnosis and immune regulation, as well as act as biotherapeutics for dental applications.

Electroactive nanoparticles, such as piezoelectric (mechanical) and pyroelectric (thermal)-based nanoparticles, have the potential to eradicate infections and promote new tissue growth. Preliminary investigations have been conducted to explore the potential usage of these smart nanoparticles; however, deeper research is necessary to better understand their clinical applications, particularly their toxicological aspects.

The development of smart nanoparticles could represent the next generation of dental materials. While they offer significant promise, extensive research is needed to ensure their safety and effectiveness in clinical practice.

## 6. Summary

Antimicrobial nanoparticles have garnered significant attention for their remarkable antibacterial and antioxidant properties in dental applications. These nanoparticles possess the potential to control bacterial growth, inhibit biofilm formation, and alleviate oxidative stress. Various dental materials, including composite resins and glass ionomer cements, have incorporated these nanoparticles to prevent dental caries and periodontal disease. Laboratory studies have demonstrated the efficacy of antimicrobial nanoparticles in enhancing dental material performance and promoting oral health. However, evidence-based clinical studies are essential to substantiate these benefits in patient care and ensure their safe and effective application in real-world settings. Comprehensive clinical research is crucial for translating promising laboratory findings into practical dental treatments that can improve patient outcomes.

## Figures and Tables

**Table 1:** Biological properties, potential applications, and limitations of antimicrobial nanoparticles.

Antimicrobial Nanoparticles	Biological Properties	Potential Applications	Limitations
***Metals***			
Silver		Treatment of dental caries	Toxicity
		Treatment of Periodontal diseases	Biodistribution and clearance
		Coating to prevent peri-implantitis	Environmental pollution
		Coating to prevent denture stomatitis	
		Disinfection in endodontic therapy	
Gold		Treatment of dental caries	Toxicity
		Treatment of periodontal diseases	
		Coating to prevent peri-implantitis	
		Coating to prevent denture stomatitis	
Copper	Promote angiogenesis	Treatment of dental caries	Toxicity
	Promote osteogenesis	Treatment of periodontal diseases	
		Coating to prevent peri-implantitis	
***Metal oxides***			
Zinc oxide	Anti-cancer	Treatment of dental caries	Toxicity
		Treatment of periodontal diseases	
		Coating to prevent peri-implantitis	
		Coating to prevent denture stomatitis	
Cerium oxide	Antioxidant	Treatment of dental caries	Toxicity
		Treatment of periodontal diseases	
		Coating to prevent peri-implantitis	
		Coating to prevent denture stomatitis	
Titanium dioxide		Treatment of dental caries	Toxicity
		Treatment of periodontal diseases	
		Coating to prevent peri-implantitis	
		Coating to prevent denture stomatitis	
		Scaffolds for bone grafting surgery	
***Polymer***			
Chitosan	Antioxidant	Treatment of dental caries	Weak mechanical strength
		Treatment of periodontal diseases	
		Coating to prevent peri-implantitis	
***Polyphenol***			
Curcumin	Antioxidant	Treatment of periodontal diseases	Poor solubility
		Coating to prevent peri-implantitis	Inconsistent bioavailability

## Data Availability

Not applicable.
